# ResidueFinder: extracting individual residue mentions from protein literature

**DOI:** 10.1186/s13326-021-00243-3

**Published:** 2021-07-21

**Authors:** Ton E Becker, Eric Jakobsson

**Affiliations:** 1 Department of Molecular and Integrative Physiology, Beckman Institute for Advanced Science and Technology, University of Illinois at Urbana-Champaign, Illinois 61801 Urbana, USA; 2 Department of Biochemistry, Program in Biophysics and Computational Biology, National Center for Supercomputing Applications, University of Illinois at Urbana-Champaign, Illinois 61801 Urbana, USA

**Keywords:** Amino Acid Residue, Mutation, Point Mutation, Natural Language Processing, Text Mining, MutationFinder, Bioinformatics

## Abstract

**Background:**

The revolution in molecular biology has shown how protein function and structure are based on specific sequences of amino acids. Thus, an important feature in many papers is the mention of the significance of individual amino acids in the context of the entire sequence of the protein. MutationFinder is a widely used program for finding mentions of specific mutations in texts. We report on augmenting the positive attributes of MutationFinder with a more inclusive regular expression list to create ResidueFinder, which finds mentions of native amino acids as well as mutations. We also consider parameter options for both ResidueFinder and MutationFinder to explore trade-offs between precision, recall, and computational efficiency. We test our methods and software in full text as well as abstracts.

**Results:**

We find there is much more variety of formats for mentioning residues in the entire text of papers than in abstracts alone. Failure to take these multiple formats into account results in many false negatives in the program. Since MutationFinder, like several other programs, was primarily tested on abstracts, we found it necessary to build an expanded regular expression list to achieve acceptable recall in full text searches. We also discovered a number of artifacts arising from PDF to text conversion, which we wrote elements in the regular expression library to address. Taking into account those factors resulted in high recall on randomly selected primary research articles. We also developed a streamlined regular expression (called “cut”) which enables a several hundredfold speedup in both MutationFinder and ResidueFinder with only a modest compromise of recall. All regular expressions were tested using expanded F-measure statistics, i.e., we compute *F*_*β*_ for various values of where the larger the value of *β* the more recall is weighted, the smaller the value of *β* the more precision is weighted.

**Conclusions:**

ResidueFinder is a simple, effective, and efficient program for finding individual residue mentions in primary literature starting with text files, implemented in Python, and available in SourceForge.net. The most computationally efficient versions of ResidueFinder could enable creation and maintenance of a database of residue mentions encompassing all articles in PubMed.

**Supplementary Information:**

The online version contains supplementary material available at 10.1186/s13326-021-00243-3.

## Background

The sequence of amino acids in a protein is known to affect the functioning of the protein. Sometimes the identity of an individual amino acid may have a dramatic effect. This is true for some diseases such as sickle cell disease [1], cystic fibrosis [2], and Huntington’s Disease (repeat of a single amino acid) [3]. A major change in the function of a protein is exemplified by switching the selectivity of an ion channel between sodium and calcium[4] or inducing lithium sensitivity in an important enzyme[5]. While these cited effects are particularly dramatic, it is likely that many other effects of identities of single amino acids, or groups of amino acids, are similarly important. However, the importance may not yet be recognized. In many cases identities of individual amino acids are indicated in research papers. While databases such as UniProtKB [6] contain comprehensive complete amino acid sequences of proteins, no such comprehensive database exists of amino acid mentions in research papers, which would have the added advantage of context provided in the body of the papers. Our study presents a protocol, implemented in Python code, for identifying individual amino acid mentions in papers. The protocol may be used for searching the PMC (PubMed Central®) database for amino acid mentions and could be used in construction of a comprehensive database of such mentions.

Automated mining of biological literature for significant facts and concepts is a challenging area of bioinformatics, due to the variation in terminology and syntax [7]. Yet such mining is vital to progress, as the corpus of literature is constantly expanding, while the brains of researchers who must take prior results into account are not. Thus, we are in danger of losing, or wasting resources rediscovering, old knowledge. One particular type of knowledge whose volume has exploded in recent decades consists of the effects of individual amino acids on function of biomolecules. These effects are revealed in different forms of evidence, including variation and conservation patterns throughout protein evolution, and site-directed mutagenesis experiments.

Much of the work describing the efforts to mine literature on individual amino acids is reviewed by [8], and the reader is referred to that work for detailed background. Klein et al. [9] proposed an infrastructure for evaluation of programs that identify mutations. Much of the previous work involves identifying mutations. MutationFinder (MF) is a prime example of a tool for such identification by Caporaso et al. [10] and has been used by multiple authors [11–17]. As an example, the tool SETH integrates MF with other complementary protocols for identifying mutations and adds modules for normalization to dbSNP and UniProt [18].

Other researchers have developed their own tools and suggested standards for identifying mutations [19–32] A few researchers have developed and described the operation of tools for identifying individual amino acids without regard to mutations [33, 34].

Our goal in the present work is (1) to construct and present an open readily modifiable program for identification of individual amino acids extracted from the literature, and (2) to explore choices that affect precision, recall, and speed. The program is built on MF, with modifications to search for residue mentions rather than mutations.

This work expands the capabilities of MF in three directions. (1) It extends recognition from mutations to all residue mentions, (2) it has an expanded regular expression(regex) that covers more variations in the style of amino acid mentions that occur in scholarly articles, and therefore scores well on complete articles as well as abstracts, and (3) it includes a choice of regex (“cut”) that permits computational speedup by a factor of several hundred for finding mutation or residue mentions, with only a modest loss of recall. The user may select from a number of regexes depending on the desired type of output.

Another source of individual amino mentions in proteins is the UniProtKB database. To do a search in PMC for amino acid mentions centered around a particular protein (each UniProtKB entry is centered around a single protein) we add the protein identifiers to the filter term to search for amino acid mentions. To illustrate this, we used the search term ‘P07900’ to find papers about heat shock protein. The very first paper [35] was not mentioned in the UniProtKB entry; ResidueFinder(RF) found 13 true positive amino acid mentions in that paper alone. The UniProtKB entry includes only five mentions from four articles, none of which are in the above-cited paper.

In a more extensive evaluation of a UniProtKB search, we consider our previous work [36], in what we discovered from the Shaker channel (UniProtKB P08510). There were 299 amino acid mentions in the literature while the UniProtKB page only contains 11.

We conclude that, in at least some cases, our methods will uncover amino acid mentions that would not be revealed by searching the UniProtKB database.

## Implementation

The program utilizes the same implementation as MF, command line Python 2.7 across all platforms.

The MF code was modified in the following ways:

- Changing the basic format of the search object from a mutation to a residue.
- Accepting single digit as well as multiple-digit sequence numbers. This change improves recall, but it reduces precision substantially. We judge that this trade-off well worth while because otherwise we would be systematically throwing away mentions of a class of amino acids; that is, those near the beginning of the protein sequence.
- Production of a simplified output file. The simplified file only reports one entry for each unique residue or mutation mention in the article, rather than including each mention as a separate entry, and is thus easy to read quickly and to use for scoring the performance of the program. The fact that a residue is mentioned in an article provides a good reason to read the article, so as a default we report full statistics for unique mentions only. However, we retain as an option the ability to report all instances of a particular mention in each document, as a user choice.
- Adding regular expression (regex) files to accommodate a broad range of residue mention formats.
- A choice of regular expression files to accommodate user preferences for precision versus recall, and for recall versus computational efficiency. All regexes used in this study are provided in Additional Materials as .txt files..

We adopt a performance standard based on the full text of journal articles, in addition to the abstracts-only text that was reported in Reference [10]. The behavior of the program is tested on different sets of papers, as follows:

Paper Set I: We report a detailed performance on 20 complete articles that are obtained in PDF format randomly selected from 1278 papers describing voltage gated potassium channel proteins [36]. These 20 papers were examined in exhaustive detail for all mentions of specific residues. Automated performance was compared to manual inspection informed by deep familiarity on the part of the authors with this protein family.

Paper Set II: Development set, these were abstracts used by MutationFinder to develop that program [10].

Paper Set III: Test set, these were abstracts used by MF to do a blind test on the performance of MF [10].

Paper Set IV: 100 complete articles were derived by the following procedure: All PMCID entries in the PMC database were searched automatically using the keywords “amino acid”, “residue”, “motif”, “sequence”, “protein”. Entries returned by one or more of those keywords were shuffled into a random order. They were scanned manually for at least one mention of an amino acid until 100 amino acid mentioning articles were retrieved. This required manually searching 489 articles. (We found that the keyword screening was necessary to reduce the search space. Without such screening, only 3 out of 100 randomly selected PMCID’s were found to represent research articles mentioning amino acids.)

Paper Set V: 20 randomly selected articles from Set IV to measure the relative performance of a full count of all mentions of residues in a paper, as opposed to verification that a residue is mentioned at least once in a paper.

Paper Set VI: The corpora underlying Verspoor et al. [34], for purpose of comparing our results with theirs.

Other data sets in addition to Paper Sets I through VI were also tested and the results can be seen in Additional Materials. Also, for Paper Set I, the exact reasons and samples of False Negatives, True Positives, and False Positives are shown in Additional Materials, in the highlights tab of Additional File 2.xlsx.

The regexes tested were as follows:

- Regex 1 was analogous to the regex used in MF. MF finds the equivalent of a residue identifier followed immediately by a number indicating the location in the protein immediately followed by a second residue (the mutation) identifier. Regex 1 in RF is identical except that the second residue identifier is not required for a positive identification. Regex 1 was tested in Paper Sets I, II, and III.
- Regex 2 is Regex 1 plus allowing a space between the first residue identifier and the number indicating the location in the protein. Regex 2 was tested in Paper Set I. Recall was improved and precision degraded relative to Regex 1. Performance was in every way intermediate between Regex 1 and Regex 3. Results for Regex 2 available in additional data.
- Regex 3 adds an allowance of essentially any expression that includes a capitol letter followed by a number with no consideration for what is immediately before the capitol letter or after the number. Regex 3 was tested in Paper Sets I, II, III, IV, V, and VI. A modified Regex 3 voided out 3 individual patterns to avoid excessive redundancy of return when reporting full count was tested on Paper Sets V and VI.
- “Cut” versions of several of the above, formed by removing all components of each regex except those which reflect the most common nomenclature for residue and mutation mention. The “cut” regexes provide a several hundredfold speedup with only modest penalty in recall. Cut versions were tested on Test Sets I, II, III, and IV. We also created and tested a cut version of the MF regex and tested that version on the published MF development and test sets.

This simulation protocol is designed to explore the tradeoffs in speed, recall, and precision resulting from choices of how many and which expressions to include in each regex. Compute time for each run of the program is, to a good approximation, directly proportional to the length of the documents searched and to the number of expressions in the regex used to search the documents. Because the expressions chosen for the “cut” versions of each regex are selected to be the most-used expressions, the “cut” versions provide an advantage not only in speed but also precision. On the other hand, the more complete regexes, while causing the program to run more slowly, provide greater recall, albeit at the cost of returning more false positives. As a specific example of speedup, the cut version of Regex 3 has six expressions whereas the full Regex 3 has 1518. The run times for a full text corpora (from Table 1, lines 21 and 22) are 259m12.181 s for Regex 3 vs. 48.696 s for Regex 3 cut for a speedup ratio of 317, while the ratio of the number of expressions is 1518/6 or 253.

Table1 shows the performance of the major paper sets and regexes used. The trade-offs between using a full regex versus a “cut” regex are shown; the latter runs up to a couple hundred-fold faster with only a minimal loss of recall. The value of running full text articles versus just abstracts through the program is shown; the gain of recall from processing full papers more than compensates for the increased computer time. These trade-offs are illustrated with processing six different sets of papers. The importance of providing this table allows a user to have an informed decision of which regex will deliver the optimal results for their desired needs, and the extent to which that choice might vary based on the particular set of documents to be processed. More details relevant to understanding this table are provided in the text.

**Table 1:** Performance of different regexes with different datasets

	Program and Version	Paper Set	TP	FP	FN	P	R	F_1_	F_2_	time
1	MF(Full text) (20) (Mutations)	I	66	3	102	0.957	0.393	0.557	0.445	13m22.452s
2	MF cut(Full text)(20)	I	66	3	102	0.957	0.393	0.557	0.445	0m1.600s
3	MF (Only Abstracts)(20)	I	9	0	5	1.000	0.643	0.783	0.692	0m33.612s
4	MF cut(Only Abstracts)(20)	I	9	0	5	1.000	0.643	0.783	0.692	0m0.120s
5	RF Regex 1(Full text)(20)	I	144	64	264	0.692	0.353	0.468	0.391	11m21.468s
6	RF 1 (Only Abstracts) (20)	I	15	13	8	0.536	0.652	0.588	0.625	0m32.240s
7	RF Regex 3 (Full text)(20)	I	385	602	23	0.390	0.944	0.552	0.735	56m30.868s
8	RF Regex 3 cut(Full text)(20)	I	370	569	38	0.394	0.907	0.549	0.720	0m8.896s
9	RF 3 (Only Abstracts)(20)	I	22	27	1	0.449	0.957	0.611	0.780	2m5.648s
10	RF 3 cut(Only Abstracts)(20)	I	21	27	2	0.438	0.913	0.592	0.750	0m0.440s
11	MF devo set (Only Abstracts)	II	201	4	26	0.980	0.885	0.931	0.903	4m51.408s
12	MF devo set cut (Only Abstracts)	II	175	0	52	1.000	0.771	0.871	0.808	0m0.748s
13	MF test set (Only Abstracts)	III	305	13	64	0.959	0.827	0.888	0.850	8m27.164s
14	MF test set cut (Only Abstracts)	III	257	0	112	1.000	0.696	0.821	0.741	0m1.208s
15	RF Regex 1(Full text)(100)	IV	661	378	520	0.636	0.560	0.595	0.573	56m7.653s
16	RF Regex 1 cut(Full text)(100)	IV	566	373	615	0.603	0.479	0.534	0.500	0m16.747s
17	RF Regex 1(no bib)(100)	IV	661	338	520	0.662	0.560	0.606	0.577	43m21.403s
18	RF Regex 1 cut(no bib)(100)	IV	561	341	620	0.622	0.475	0.539	0.499	0m13.200s
19	RF 1 (Only Abstracts) (100)	IV	59	12	45	0.831	0.567	0.674	0.606	1m15.552s
21	RF Regex 3 (Full text)(100)	IV	1030	2969	151	0.258	0.872	0.398	0.590	259m12.181s
22	RF Regex 3 cut(Full text)(100)	IV	878	2938	303	0.230	0.743	0.351	0.514	0m48.696s
23	RF Regex 3(no bib)(100)	IV	1027	2407	154	0.299	0.870	0.445	0.629	190m45.317s
24	RF Regex 3 cut(no bib)(100)	IV	876	2385	305	0.269	0.742	0.394	0.549	0m37.872s
25	RF 3 (Only Abstracts)(100)	IV	81	143	23	0.362	0.779	0.494	0.633	5m31.332s
26	RF 3 cut(Only Abstracts)(100)	IV	71	142	33	0.333	0.683	0.448	0.564	0m1.379s
27	RF 1 Single Count(20)	V	152	53	141	0.741	0.519	0.610	0.552	
28	RF 1 Full Count(20)	V	590	240	766	0.711	0.435	0.540	0.472	
29	RF 3 Single Count(20)	V	212	607	44	0.259	0.828	0.394	0.575	
30	RF 3 Full Count(20)	V	1199	3657	157	0.247	0.884	0.386	0.583	
31	Results from Verspoor et al.	VI	2463	412	245	0.857	0.910	0.882	0.898	
32	R3 Single Count Verspoor data	VI	1345	1230	31	0.522	0.977	0.681	0.832	
33	R3 Full Count Verspoor data	VI	3123	4558	94	0.407	0.971	0.573	0.760	

All regexes as well as the program itself are downloadable from Additional Material as well as from SourceForge.net.

The performance test was based on the following rules:

True Positive (TP) - the program returns a mention of a residue and the residue is in fact mentioned.

False Positive (FP) - the program returns a mention of a residue, but the residue was not mentioned.

False Negative (FN) - the program doesn’t return a mention of a residue, but the residue was mentioned.

There also is such an entity as a True Negative (the program does not return a mention and the residue was not mentioned) but in the context of this study that was deemed not a useful concept and it is not used in evaluating performance.

We evaluate the performance by considering RF as a binary classifier; i.e., each residue is either found, or not found, in a particular article. A traditional performance measure for binary classifiers is F-measure [37], as displayed below:

$${F}_{\beta }=\frac{\left({\beta }^{2}+1\right)\bullet precision\bullet recall}{\left({\beta }^{2}\bullet precision\right)+recall}$$

For information retrieval from text, it has been suggested that recall should be weighted more heavily than precision, leading to F_2_ rather than the traditional F, also called F_1_. We note that the F-measure provides for flexibility in the importance attached to precision relative to recall through adjustment of the parameter β. When β equals 1(a common choice) there is a balance between precision and recall. The higher the value of β the more importance is placed on recall, and vice versa. At the extreme, when β equals zero, the expression for F reduces to the precision end, while when β equals infinity, the expression for F reduces to the recall end. We tend to favor β > 1 because false positives can readily be identified and filtered out by subsequent manual inspection of papers of exceptional interest, whereas false negatives are permanently lost.

The choice of regex within RF should be considered a term-weighting scheme [38]. Each amino acid representation included in a regex represents a high weight establishment for that representation; each possible amino acid representation not included represents a zero weight for that representation.

## Results and Discussion

The results of a large number of investigations are summarized in Table 1. The results in Table 1 are based on the spreadsheet “Additional file 2.xlsx”, provided in Additional Material. A readme file for navigating Additional file 2.xlsx is provided in Additional File 1.doc The regexes used in the calculations underlying Table 1 are given in Supplementary Material. Specifically, the MutationFinder regex is given in Additional File 3.txt; A cut version of the MutationFinder regex is given in Additional File 4.txt; Regex 1 is given in Additional File 5.txt; A cut version of Regex 1 is given in Additional File 6.txt; Regex 2 is given in Additional File 7.txt; A cut version of Regex 2 is given in Additional File 8.txt; Regex 3 is given in Additional File 9.txt; a cut version of Regex 3 is given in Additional File 10.txt.

For a detailed guide to interpreting analysis for one of the papers in the study, we choose Paper #6 in Tab “highlights” in the Spreadsheet “RF Excel Supplement” available as Additional File 2.xlxs This paper is PMID 10370099 Höllerer-Beitz, Gerhild, Roland Schönherr, Michael Koenen, and Stefan H. Heinemann. “N-terminal deletions of rKv1. 4 channels affect the voltage dependence of channel availability.“ *Pflügers Archiv* 438, no. 2 (1999): 141–146. We show results from analyzing the full text of the paper. This text has 6 residue mentions based on a close manual inspection.

Columns B-D show results from original MutationFinder. Two mutations are found, four residue mentions are not found (as expected, because MutationFinder does not look for mention of residues not associated with mutations). Columns E-G show results from a “cut” version of the MutationFinder regex where it is shown that the speedup of the “cut” version is achieved at no cost in recall.

Columns H-J show results from Regex 1, revealing that 4 residue mentions are found, 2 residue mentions are not found. Columns K-M for the “cut” version of Regex 1 shows that only 2 of the 6 residue mentions are found. For both the full and cut versions of Regex 1, one false positive is returned.

Columns N-S show that for both the full and cut versions of Regex 2, 4 of the 6 residue mentions are found, and that one false positive is returned.

Columns T-Y show that for both the full and cut version of Regex 3, all 6 of the residue mentions are returned (for a recall of 100 %) but that 9 false positives are also returned.

Column AD provides annotation giving the nature of the false positives (bibliography, equipment description, etc.).

Column AE shows the context in the paper for all the returned expressions, both true and false positives.

Other papers mentioned in the spreadsheet may be interrogated in analogous fashion.

In order for the reader to evaluate the timings in Table 1 we note that the computer used an Ubuntu operating system, the processor was an AMD Athlon 64 × 2 at 1GHz, with access to 2.8 GB RAM. Because of the modest performance of the machine, we expect the timings to be better on newer machines.

The first set of investigations summarized in Table 1 was done on a set of 20 papers all of which were on potassium channels (a particular interest of ours) that mention individual amino acids, randomly selected from a much larger set of over a thousand research articles. The first two rows show the performance of MF on this set. (The regex for MF is provided in Additional File 1.txt) We see from rows 1 and 2 that the full text provides a significantly more severe test of the program than abstracts alone, especially with respect to recall. Comparison of rows 2 and 4 with rows 1 and 3 shows that it was possible to improve the efficiency of MF dramatically without compromising performance at all (for this particular set of papers) by eliminating from the regex all patterns except the most common. This is described in the Implementation section and is provided in detail in the Additional Material 2.xlxs. This would be recommended for using MF to process a large number of papers, with the caveat that the particular regexes to be removed should be checked for other sets of papers than this particular set.

Comparing rows 1 and 3 with rows 5–6 shows the effect of simply using the analogous filters of MutationFinder in ResidueFinder (Regex 1). This comparison reveals that there are many more formats for a residue mention than for a mutation mention, so the performance of RF with this regex is statistically far worse than the performance of MF for the same set of papers.

To compare rows 5–6 with rows 7 and 9, one should understand the differences between Regex 1 and Regex 3. This expansion was in two steps, (1) addition of two patterns that were responsible for the largest number of false negatives with Regex 1, and step (2) for each of the pattern addition of a version that includes a space between the amino acid identifier and the location number. While standard nomenclature suggests not to include a space [39] we found numerous examples of insertion of a space in the text, warranting inclusion of that variation in the regex. There are different classes of errors that cause a FP. These error types are broken down in Table 2 whereas the two different types of FN are shown in Table 3.

Table 2 Gives the classes of error resulting in FPs, for Paper Set I. Since some mentions leading to FPs have more than one contributing cause, the total number of incidences of causes adds up to more than the total number of FP identifications.

Table 3 Shows causes of FN errors in Paper Set 1 as residue not found because they were embedded in a non-readable image or the regex did not have the correct pattern match to identify as a residue. In principle the FN errors in the images could be overcome with OCR technology.

**Table 2:** Classes of FP Errors in RF using Regex 3 on full text

Type	Number	Percent
Motif Name	94	15.1
Equipment/Substrate	73	11.8
Bibliography/Reference	180	30
Protein or Ground Name	104	16.7
Short Name	12	1.9
PDF2text Artifact/proximity	26	4.2
Formula/other nomenclature	132	21.3

**Table 3:** Classes of FN errors in RF using Regex 3

Type	**Number**	**Percent**
In an image	15	68.2
Regex not found	7	31.8

In rows 8 and 10 we see the results of introducing a “cut” version of Regex 3. The cut version is created by eliminating all notations except (1) single capitol letter amino acid code or three letter amino acid followed with no space in front of the location number, (2) single capitol amino acid letter or three letter amino acid followed by one space and then the location number. MF and RF regexes include many other possibilities that turn out to be relatively rare, so the “cut” version runs hundreds of time faster with only minor degradation of performance.

Comparing rows 11 with 13 and rows 12 and 14 show the results of creating a “cut” version of MutationFinder as measured by performance on the development and test corpora used by those authors. We see that MF, as is the case with RF, is dramatically improved in speed with only minor degradation of performance as indicated by precision, recall, and F-measure. Note that these corpora are represented by abstracts.

Beginning with row 15 and continuing through row 26, we introduce results on a random set of 100 papers. The papers were screened to mention “amino acid” or “residue” in either a MeSH or text in a search of PubMed Central. Following screening, the 100 were chosen from all that passed the screen by a random number generator operating on PMC identifying numbers. By comparing rows 5–10 with 15–26 we show the effects of moving from a set of papers selected randomly from a particular field (potassium channels, rows 5–10) to a set of papers selected randomly from all fields of protein science (rows 15–26). We find that performance on the randomly selected papers does not seem systematically different from the performance on the potassium channel papers. The fact that the K + channel papers were selected by intensive manual search provided an opportunity to estimate the recall for the key word search for “amino acid” and “residue”. We found that this key word search retrieved 265 out of 329 K + channel papers that we had previously ascertained contained amino acid mentions, for a recall of 0.805. Based on other comparisons between the K + channel set and the more general set, we see no reason to expect the recall for the more general papers to be significantly different from the K + channel papers.

Rows 15–19 show results for variants on Regex 1 (similar to MutationFinder) and Regex 3, which is somewhat streamlined as described in Implementation.

By doing pairwise comparison between rows 15–16, 17–18, 21–22, and 23–24 we see that the “cut” version of each regex suffers only marginal degradation of performance compared to the full versions but is speeded up by a factor of hundreds, in most cases over 200-fold. Thus, we would recommend the “cut” version to process a very large number of papers.

By pairwise comparison between rows 15 and 17, 16 and 18, 21 and 23, and 22 and 24, we see the effect of removing the bibliography from the text. We find processing time reduced by a factor of approximately ¾, a moderate deterioration in precision, and essentially no deterioration in recall. The underlying phenomenon is that the regex found almost no true positives in the bibliography, so perusing the bibliography is essentially wasted computer time. Also, culling the bibliographies from PMC articles is readily automatable, so this is recommended in this context. On the other hand, for articles not available in PMC, the variety of formats makes culling the bibliography more difficult.

Rows 19 and 25 show performance on abstracts only. Statistically the performance on the abstracts looks better than performance on the full texts, but this is misleading because there are many more amino acids mentions in the text than in the abstract. Comparing row 15 with 19 and 21 with 25 shows that inspecting only the abstracts misses over 90 % of the amino acid mentions. By comparing 15 with 16, 17 with 18, 21 with 22, 23 with 24, and 25 with 26, we see that the “cut” version of each regex loses only 12.3–15.1 % of true positive amino acid mentions but achieves a much better speedup than keeping the full regex and scanning only abstracts. Because document preparation for PMC papers is only marginally more effort than the abstracts alone, and because an increasing fraction of papers are available in PMC, it does not seem to us to be a useful strategy to scan abstracts alone unless inspecting collections that include a large fraction of papers not available as PMC.

Rows 27 through 30 represent calculations designed to compare our work with that of Verspoor et al. [34], which motivates us to shift from evaluation based on “at least one mention of an amino acid in a paper” to “all mentions of an amino acid in a paper”. In order to facilitate manual verification of program performance by the “all mentions” criterion, we randomly chose 20 papers out of the set of 100 that was the subject of the calculations in rows 15 through 26. Comparison of row 21 with 29 shows that RF’s performance on the 20 papers was essentially the same as on the 100, suggesting that the 20 is a representative sample. Comparison of row 29 with 30 shows that the statistical performance of our program by the “at least one mention” versus the “any mentions” criteria was essentially the same. We note that the program output includes all mentions for future reference regardless of whether the performance is calculated on an “at least one mention” or an “any mentions” basis. With this equivalence in mind, we apply RF to the Verspoor et al. [34] corpus of papers, with the results shown in row 32 (single count) and 33 (full count). Comparing row 27 to row 31 and row 28 to 33 we see that our program performs significantly better on the Verspoor et al. corpus than on randomly selected papers. We ascribe this to the mode of selection of the Verspoor et al. papers, which were chosen to have as subject proteins for which PDB structures were known. We hypothesize that papers thus selected will also have more standardized nomenclature for amino acids, and therefore be more amenable to an automated search for amino acid mentions by regular expressions than our papers selected by keyword search. Comparing rows 30 and 31, we see that RF, while performing better on the Verspoor corpus than on randomly selected papers, does not perform as well as the Verspoor program based on the F1 measure. Close inspection shows that the difference is due to the larger number of false positives, and hence lower precision, from RF. On the other hand, RF showed better recall than Verspoor, so that the F2 measure, which emphasized recall, showed the two programs to be closely matched.

## Conclusions

RF is a robust command line-driven Python program for finding mentions in the scientific literature of individual amino acids contained in peptides and proteins. The added regexes allow a user to choose the combination of computational efficiency, precision, and recall that is most appropriate for that user’s needs.

The very limited “cut” regex allows an extremely rapid result (cutting the compute time several hundred-fold compared to the full regex) with a relatively small increase in false negatives. Using our relatively modest 1 GHz processor, we extrapolate from our admittedly limited sample that the time using our “cut” regexes to process a million abstracts would be approximately 3.3 CPU-hrs, and a million full texts would be approximately 168 CPU-hrs. Assuming approximately 24 million readily accessible abstracts and articles at the time of this writing and approximately one million per year being added, a first draft mutation and residue mention database could be created using “cut” regexes at a cost of 4027 CPU-hrs and could be maintained at an annual cost of 168 CPU-hrs. These numbers are very approximate, but their order of magnitude indicates clearly that such a project would be feasible.

Examples provided in the body of this paper suggest that search of the sort we have developed can provide a more comprehensive listing of residue mentions for a particular protein than is available in the UniProtKB database. To extend this potential capability further we are extending the approach in this paper to analyze Pubmed-indexed articles for linked protein and residue mentions and will report on this work in the future.

The approach described in this paper is limited in that it will never achieve both perfect precision and also perfect recall. Rather we are left with tradeoffs between computational efficiency, precision, and recall, all dependent on the choice of expressions in the regex. The fundamental limitation is that the approach does not consider context, except in a very limited way, in contrast to a careful human reader, who does understand context. Thus, assessing performance of the software still involves human review of the papers in the corpora. At the end of the day for example, it is only by a human understanding the context of the scientific paper that one can know whether the expression “T7” in a paper refers to “threonine in position 7”, as opposed to a reference to Bacteriophage T7. Thus, to make significant further progress it will be necessary to embed ResidueFinder in an Artificial Intelligence environment that can make these distinctions. Beyond this (relatively) straightforward task of distinguishing false positives from true positives will be the greater challenge of linking each true positive to entries in databases that provide additional biological meaning and global context, especially UniProt but also others.

## Availability and requirements

- **Project name**: ResidueFinder.
- **Project home page**: https://sourceforge.net/projects/residuefinder/.
- **Operating system(s)**: Platform independent.
- **Programming language**: Python 2.7.
- **License**: MIT (Slightly modified to extend to regexes).
- Permission is hereby granted, free of charge, to any person obtaining a copy of this software and associated documentation files (the “Software”), to deal in the Software without restriction, including without limitation the rights to use, copy, modify, merge, publish, distribute, sublicense, and/or sell copies of the Software (including regexes), and to permit persons to whom the Software is furnished to do so, subject to the following conditions:
- The above copyright notice and this permission notice shall be included in all copies or substantial portions of the Software.
- THE SOFTWARE IS PROVIDED “AS IS”, WITHOUT WARRANTY OF ANY KIND, EXPRESS OR IMPLIED, INCLUDING BUT NOT LIMITED TO THE WARRANTIES OF MERCHANTABILITY, FITNESS FOR A PARTICULAR PURPOSE AND NONINFRINGEMENT. IN NO EVENT SHALL THE AUTHORS OR COPYRIGHT HOLDERS BE LIABLE FOR ANY CLAIM, DAMAGES OR OTHER LIABILITY, WHETHER IN AN ACTION OF CONTRACT, TORT OR OTHERWISE, ARISING FROM, OUT OF OR IN CONNECTION WITH THE SOFTWARE OR THE USE OR OTHER DEALINGS IN THE SOFTWARE.

## Supplementary Information

Additional file 1 This document guides the reader through the interpretation of Additional file 2.

Additional File 2 Computed Performance Data. This spreadsheet directly shows the numbers underlying the tables presented in the manuscript, including the PMID’s for documents analyzed.

Additional file 3 Part of archive containing the regexes used and analyzed in this study. This part of the archive is the MutationFinder regex. It is also in the SourceForge web site.

Additional File 4 Part of archive containing the regexes used and analyzed in this study. This part of the archive is the cut version of the MutationFinder regex. It is also in the SourceForge web site.

Additional file 5 Part of archive containing the regexes used and analyzed in this study. This part of the archive is the full version 1 RF regex. It is also in the SourceForge web site.

Additional file 6 Part of archive containing the regexes used and analyzed in this study. This part of the archive is the cut version of the version 1 regex. It is also in the SourceForge web site.

Additional file 7 Part of archive containing the regexes used and analyzed in this study. This part of the archive is the full version 2 RF regex. It is also in the SourceForge web site.

Additional file 8 Part of archive containing the regexes used and analyzed in this study. This part of the archive is the cut version of the version 2 regex. It is also in the SourceForge web site.

Additional file 9 Part of archive containing the regexes used and analyzed in this study. This part of the archive is the full version 3 RF regex. It is also in the SourceForge web site.

Additional file 10 Part of archive containing the regexes used and analyzed in this study. This part of the archive is the cut version of the version 3 regex. It is also in the SourceForge web site.

## Data Availability

The datasets used and analyzed during the current study are available from the corresponding author on reasonable request.

## Abbreviations

PMC: PubMed Central®
MF: MutationFinder
RF: ResidueFinder
regex: Regular expression
FN: False Negative
FP: False Positive
TP: True Positive
P: Precision
R: Recall
OCR: Optical Character Recognition
