# Supplementary material for: ResidueFinder: extracting individual residue mentions from protein literature
Source: J Biomed Semantics. 2021 Jul 21;12:14. doi: 10.1186/s13326-021-00243-3 (PMC8293528; doi:10.1186/s13326-021-00243-3)
Supplement: Supplementary file 1 — This document guides the reader through the interpretation of Additional file 2. [file 13326_2021_243_MOESM1_ESM.docx]

Readme for Navigating Spreadsheet Showing Comprehensive Results for this Paper in Additional

Additional material as an Excel document is provided to get a more complete understanding of the different studies underlying this paper. Much of the additional material in the excel file is for those who want to repeat the results, use the different data sets for their own use, or examine more deeply what was done. It is often in a shorthand and contains extensive scratch work meant for quick calculations or verification of data information. A few of the tabs were produced over a few months of time. Many of the tabs are semi-self-explanatory but we will describe each tab here.

TAB HIGHLIGHTS; TP (total positives) highlighted in green, FP (false positives) highlighted in red, FN (false negatives) highlighted in yellow, are shown from paper set I for each regex 1-3 and their corresponding “cut" versions. For all instances an example (if more than 1 instance) is pasted directly from the article, or reason for it being found is entered. At the bottom of the sheet is contained many of the calculations for performance. Near the bottom of the sheet on the right side are the highlights of the other tabs in a table,

TAB TABLES; This tab contains the tables used in the manuscript plus a few other tables and calculations. It contains the totally 100 random PMC ID’s of which only 3 contained amino acid mentions. It also contains the exact keyword search links and iterations used to acquire 100 articles that each contain at least one amino acid mention.

TAB OTHGOLDEV; This is the development set, paper set II, from Caporaso with results shown run with MF. Some of the FN reasons are pasted into the rows.

TAB OTHGOLDTES; Paper set III is the test set from Caporaso.

TAB ALL AA; This is the detailed method of the iterations of the keyword search, the resulting random ID screened to contain amino acid mention(s) was added to the list on the far left. It was also noted if any mention(s) were in abstracts, if not then body of papers only.

TAB AA ART; Paper set IV, the 100 random, contains all the calculations of multiple regexes, multiple parts of the articles edited as described at the top row.

TAB KCNAPMC; This is the list of manually collected articles with mentions of potassium channel amino acids, a study done in 2011 and included in the Ph.D. thesis of A.E. Becker but not previously published in a peer-reviewed journal. All entries with a PMC ID were extracted and then verified if they were found in the keyword search.

TAB UNIPROT; These results are from a study that looked at 11 random Uniprot entries with the aa mentions and the reference papers extracted for each. Different regexes were used to discover how many of the mentions were found from the article references.

TAB HSP90 SH; One of the selected random 11 UniProt entries, HSP 90 (P07900), was used to illustrate the advantage of our method of finding amino acid mentions over depending on UniProt entries. We put “P07900” as a search term in PMC database and find at this writing as the number #4 hit the paper “Ciglia E, Vergin J, Reimann S, Smits SH, Schmitt L, Groth G, Gohlke H. Resolving hot spots in the C-terminal dimerization domain that determine the stability of the molecular chaperone Hsp90. PLOS one. 2014 Apr 23;9(4):e96031”. This paper is not mentioned in the UniProt entry for this protein. Our method using Regex 3 found 13 amino acid mentions, none of which are mentioned in the UniProt entry.

This tab shows the results of aa mentions of the Uniprot entry “P08510" to compare to the list in our next tab.

TAB P08510; This is the list of aa mentions of "P08510" from reference 48 in the main paper to show how many mentions we know are in articles as compared to those mentioned in the Uniprot entry in the previous tab. It is seen that our search revealed 299 amino acid mentions as opposed to 11 amino acid mentions in UniProt.

TAB VERSPOOR; Paper set V Verspoor et al. corpora run with regex 3 and calculations of single count and full count from the numbers found in the corpora. A side study not included in manuscript shows tables and graphs discovering the values if a graduated higher F_ß_ is calculated.

TAB FULCOUNT20; This is 20 articles, paper set VI, randomly pulled from the random 100 for tallying of every single mention recorded for calculation of performance if all mentions were included. This was to show whether there was any difference in calculation performance if we counted every single mention as opposed to counting only one mention per paper per amino acid. The answer is that there is no significant difference in performance.
